# Transcriptional Regulation in Roots by Bacteria With 1‐Aminocyclopropane‐1‐Carboxylate Deaminase Enzymes for Drought Tolerance and Post‐Stress Recovery

**DOI:** 10.1111/ppl.70733

**Published:** 2026-01-02

**Authors:** William Errickson, Bingru Huang

**Affiliations:** ^1^ Department of Agriculture and Natural Resources Rutgers University New Brunswick New Jersey USA; ^2^ Department of Plant Biology and Pathology Rutgers University New Brunswick New Jersey USA

**Keywords:** bacteria, drought, grass, roots, transcriptome

## Abstract

Plant growth‐promoting rhizobacteria (PGPR) that can break down 1‐aminocyclopropane‐1‐carboxylate (ACC), an ethylene precursor, by ACC deaminase enzymes (ACCd) to reduce ethylene production in plants may enhance plant tolerance to drought stress. This study aimed to identify genes in plant roots regulated by ACCd‐bacteria under drought stress and re‐watering and to determine major molecular factors and associated metabolic pathways for ACCd bacteria‐enhanced drought tolerance and post‐stress recovery in creeping bentgrass ( *Agrostis stolonifera* ). Transcriptomic analysis was performed in root tissues from plants inoculated with a novel strain of ACCd‐producing bacteria, *Paraburkholderia aspalathi* “WSF23,” under well‐watered conditions, 35 days of drought stress, and 15 days of re‐watering. ACCd bacteria inoculation resulted in differential expression of 53 genes under drought stress. Genes up‐regulated in inoculated roots during drought stress included SUMO (small ubiquitin‐like modifier) protease *OTS1*, an alcohol dehydrogenase (*ADH2*), desiccation‐related protein (DRP) gene *pcC‐13362*, cell wall structure and elasticity (*TBL27*), and antioxidant metabolism (*DJ‐1C* and *1CYSPRXA*). For post‐drought recovery, inoculated plants differentially expressed 160 genes, including up‐regulation of DNA repair (*RAD6*), signal transduction (*WRKY72*), root growth and development (*D10*, *WRKY74*, *ERF3*), nitrogen transport (*DUR3*), and osmoregulation (*CIPK23*), as well as up‐regulation of carotenoid biosynthesis pathways. These findings help to explain the molecular mechanisms associated with ACCd bacteria‐mediated drought stress tolerance and post‐drought recovery in cool‐season perennial grass species, contributing to sustainable methods of reducing water use in turfgrass management.

## 1 Introduction

Abiotic stress, such as drought, causes an increase in ethylene production in plant tissues, which has been associated with the inhibition of root growth and induction of premature leaf senescence (Singh et al. 2015; Dubois et al. 2018; Naing et al. 2021; Chen et al. 2022). Strategies that can suppress ethylene production have shown evidence of improving drought tolerance. Inoculation of plants with different species of plant growth‐promoting rhizobacteria (PGPR), including *Paraburkholderia* and *Bacillus* , has resulted in the promotion of drought tolerance through diverse biological functions or modes of action (Glick 2014; Errickson and Huang 2019; Naing et al. 2021). PGPR that produce deaminase enzymes have demonstrated efficacy in improving drought stress tolerance, as ACC deaminase enzymes (ACCd) function to break down 1‐aminocyclopropane‐1‐carboxylate (ACC), a precursor of ethylene, into ammonia and α‐keto butyrate before ACC is converted into ethylene (Glick 2005). This can mitigate the impacts of stress‐induced ethylene production, including premature leaf senescence and reduced root growth. For example, ACCd bacteria, *Paraburkholderia aspalathi* , enhanced tiller production, root growth, whole‐plant tolerance to drought stress, and post‐stress recovery by suppressing drought‐induced ethylene production in creeping bentgrass ( *Agrostis stolonifera* L.) (Errickson et al. 2023).

Drought‐tolerant and drought‐susceptible genotypes of a given plant species often exhibit differential gene expression related to drought tolerance, suggesting mechanisms by which the drought‐tolerant genotypes achieve this level of improved tolerance (Tan et al. 2022). Novel approaches to stimulate stress resistance in turf have included exogenous applications of compounds, such as proline, gamma‐aminobutyric acid (GABA), and nitric oxide (NO), to stimulate stress‐defensive gene expression and antioxidant activity (Jiang et al. 2023; Jiang 2023). PGPR inoculation may also induce changes in gene expression that contribute to improved drought tolerance, as PGPR can exhibit other plant growth‐promoting traits in addition to ACCd activity, including stimulation of other hormones, antioxidant activity, and nutrient mobilization (Lata et al. 2024). The promotion of drought tolerance in cool‐season turfgrass addresses a primary objective of sustainable turfgrass management through the reduction of irrigation requirements and subsequent water use (Wang, Olsen, et al. 2023). Symbiotic interactions between plants and endophytic bacteria may represent novel strategies to improve plant growth while reducing water use (White et al. 2021; Chang et al. 2023). While the influence of beneficial microbial endophytes on improving abiotic stress tolerance in cool‐season grass species has been documented, there remains limited understanding of the mechanisms involved, especially considering the multitude of species of beneficial endophytes that can colonize cool‐season grasses (Hewitt et al. 2022; Roberts 2022).

Recent studies in genetic and metabolic changes in turfgrass species in response to abiotic stress have started to provide insights into the molecular basis of stress tolerance (Brown et al. 2023). Metabolomic analysis of leaves and roots of creeping bentgrass colonized by *P. aspalathi* demonstrated that improved drought tolerance and post‐stress recovery were associated with several mechanisms. Leaf tissue of inoculated plants demonstrated up‐regulation of carbohydrate, pyrimidine, and cytokinin hormone metabolism. Inoculation enhanced the accumulation of metabolites in pathways related to carbohydrate metabolism, organic acid metabolism during respiration, DNA and protein synthesis, and cellular metabolism in roots (Errickson and Huang 2023). While several physiological and metabolic studies have demonstrated ACCd bacteria's abilities to confer abiotic stress resistance, including drought in various plant species (Stearns et al. 2012; Saikia et al. 2018; Naing et al. 2021; Shahid et al. 2024), the mechanisms underlying ACCd PGPR‐enhanced drought tolerance, particularly post‐stress recovery at the molecular level, are poorly understood.

Creeping bentgrass is a cool‐season turfgrass species that is widely used on golf course putting greens and fairways. Strategies for improving abiotic stress tolerance in creeping bentgrass can help to maintain turf quality while reducing irrigation requirements, thus conserving natural resources and improving efficiency and economics for turfgrass managers. Creeping bentgrass is a stoloniferous perennial grass species that can tolerate varying degrees of drought stress and re‐grows upon re‐watering, making it a suitable model turfgrass species for conducting this investigation.

The objectives of this study were to understand which key genes and associated metabolic pathways to promote drought stress tolerance and post‐stress recovery in creeping bentgrass are regulated by ACCd bacteria *P. aspalathi* “WSF23,” a novel PGPR strain that was isolated from the roots of native grasses in the New Jersey Pine Barrens and demonstrated high levels of ACCd activity. This is a further research advancement from our understanding of physiological and metabolic effects of *P. aspalathi* on creeping bentgrass tolerance to drought stress and post‐stress recovery that have been reported in previous publications (Errickson et al. 2023; Errickson and Huang 2023). A comprehensive understanding of the mechanisms involved in ACCd bacteria‐mediated stress tolerance is essential for the development of PGPR as broadly applicable biostimulants and their widespread adoption for crop production, especially as it relates to reducing water use while maintaining crop quality.

## 2 Materials and Methods

### 2.1 Plant Growing Conditions

Creeping bentgrass (cv. “Penncross”) plants were established from vegetative sod plugs collected at the Rutgers Horticultural Research Farm II in North Brunswick, NJ, USA (40.46918734608342, −74.42450570308384). The sod plugs were divided into individual tillers, and the shoots and roots were trimmed. The tillers were soaked in 1% sodium hypochlorite solution for surface sterilization and then rinsed in autoclaved deionized water. Bunches of 10 tillers were planted into plastic containers (20 cm wide × 30 cm long × 20 cm deep) filled with calcined clay (Profile Products, Buffalo Grove, IL) that had been autoclaved for a 20‐min wet cycle to minimize contamination from other microorganisms. The containers were dispersed across four different controlled environment growth chambers (Environmental Growth Chambers, Chagrin Falls, OH) after a 30‐day establishment period in the greenhouse. The growth chamber conditions were controlled at 23/16°C (day/night temperature), 650 μmol m^−2^ s^−1^ photosynthetically active radiation of 12‐h photoperiod and 50% relative humidity. The plants were watered every 2 days and fertilized every 7 days with half‐strength Hoagland's nutrient solution (Hoagland and Arnon 1950) and were allowed to acclimate to these conditions for 7 days before the imposition of drought stress treatments.

### 2.2 Culture of Bacterial Inoculum and Inoculation of Plants

A novel PGPR strain of *P. aspalathi* was used in this study due to its high levels of ACCd activity and previous evidence of improving drought tolerance in creeping bentgrass (Errickson et al. 2023; Errickson and Huang 2023). Bacterial cultures of *P. aspalathi* “WSF23” were revived from frozen stock vials that had been stored at −80°C. The bacteria were cultured in Luria Broth (Miller) (Sigma–Aldrich) on a shaker set to 120 rpm at 23°C for 4 days. The bacterial suspensions were then centrifuged at 10,000 *g* for 5 min at 23°C, then resuspended in deionized water. The samples were centrifuged and resuspended twice to remove all Luria Broth from the preparations. The bacterial suspension in deionized water was then adjusted to an OD_600_ value of 1.0.

The bacterial inoculum was watered into the root zone of the plants at a rate of 75 mL per plant. A second dose of the inoculum was applied 24 h later. The inoculation treatments were applied to eight containers that each held six plants that were comprised of 10 individual tillers. A second set of eight containers that held six plants each was treated as a non‐inoculated control group and received only deionized water that did not contain the ACCd bacteria.

### 2.3 Drought Stress and Re‐Watering

Drought stress began 7 days after plants were first inoculated. Drought stress was applied by withholding all water for 35 days. After 35 days of drought stress, the plants were then re‐watered for 15 days for a post‐drought recovery. While the drought‐stressed plants all showed signs of desiccation after the drought stress period, all plants in every treatment group survived and resumed growth during the re‐watering period. Four containers, each of inoculated and non‐inoculated treatments, were subjected to drought stress and re‐watering treatments. Four containers of inoculated plants and four containers of non‐inoculated plants also received full irrigation and served as a non‐stress control group. The experiment was conducted as a completely randomized design with ACCd bacteria inoculation and drought stress and re‐watering treatments, each repeated in four containers.

### 2.4 Transcriptome Analysis

Root tissue was destructively sampled for RNA extraction from inoculated and non‐inoculated plants from the three different stress treatment groups (well‐watered, 35 days drought stress, 15 days re‐watering). All plants remained in the vegetative stage during the study, and all samples were vegetative. Three replicates were used for each treatment group. The roots were rinsed free of calcined clay with deionized water, and 0.25 g of fresh tissue was flash‐frozen with liquid nitrogen and ground in a sterile mortar and pestle before being transferred to a new 2‐mL microcentrifuge tube. RNA was isolated from the ground samples following the manufacturer's protocol for the QIAGEN RNEasy kit (Qiagen). Samples were sent to GeneWiz (Genewiz) for transcriptome analysis using RNA‐Seq (Wang et al. 2009). The rice ( *Oryza sativa* L.) genome was used as a reference because it is similar to the creeping bentgrass genome; however, it is much more complete due to significantly more genomic research being conducted on rice compared to creeping bentgrass.

### 2.5 Statistical Analysis, Gene Classification, and Metabolic Pathway Analysis

Differentially expressed genes (DEGs) were analyzed using DESeq2 (Love et al. 2014), with a threshold of 1.0 log2 fold change and *p* < 0.05. Individual genes were researched using the Rice Annotation Project Database (RAP‐DB) (Sakai et al. 2013) and Oryzabase (Kurata and Yamazaki 2006). Volcano plots for the different treatment groups were created using VolcaNoseR (Goedhart and Luijsterburg 2020). Functional classification of gene ontology was conducted using Panther 19.0 (Mi et al. 2019), and KEGG pathway analysis (Kanehisa et al. 2016) was conducted for up‐regulated and down‐regulated DEGs in each treatment group. Venny (Oliveros 2007) was used to create Venn diagrams and compare DEGs for each treatment group.

## 3 Results

### 3.1 DEG Regulated by ACCd Bacteria in Roots of Plants Exposed to Drought Stress

**FIGURE 1:**
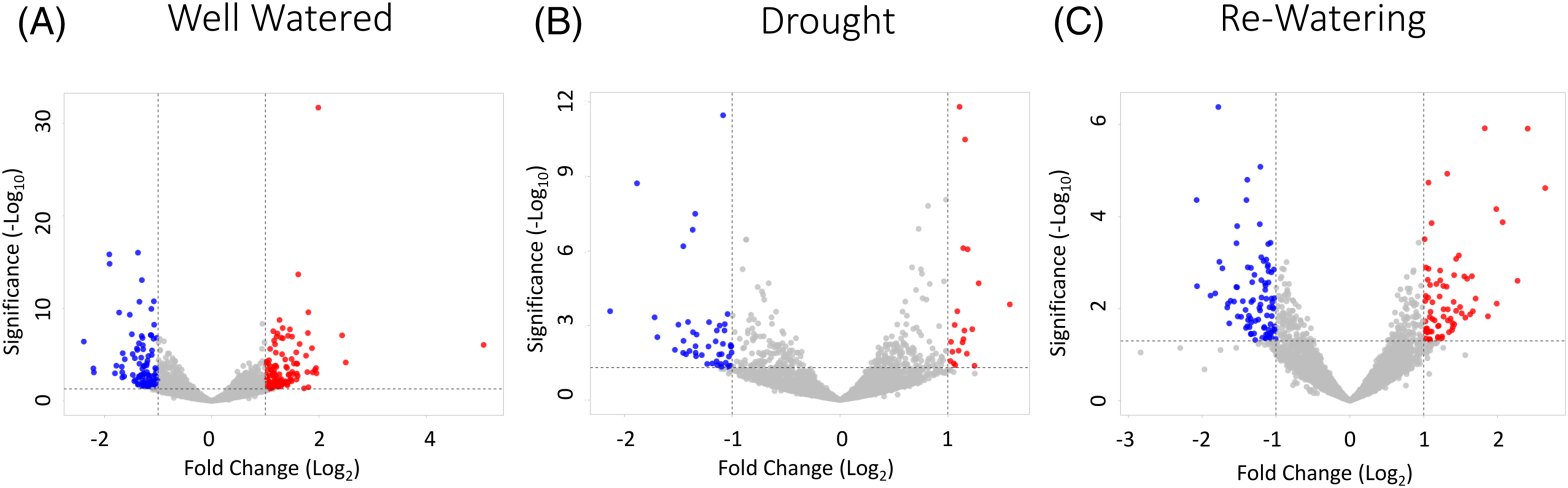
Differentially expressed genes in *P. aspalathi* “WSF23” inoculated creeping bentgrass roots under well‐watered (A), drought (B), and post‐drought recovery (C) conditions, relative to non‐inoculated creeping bentgrass roots (*p* ≤ 0.05).

**FIGURE 2:**
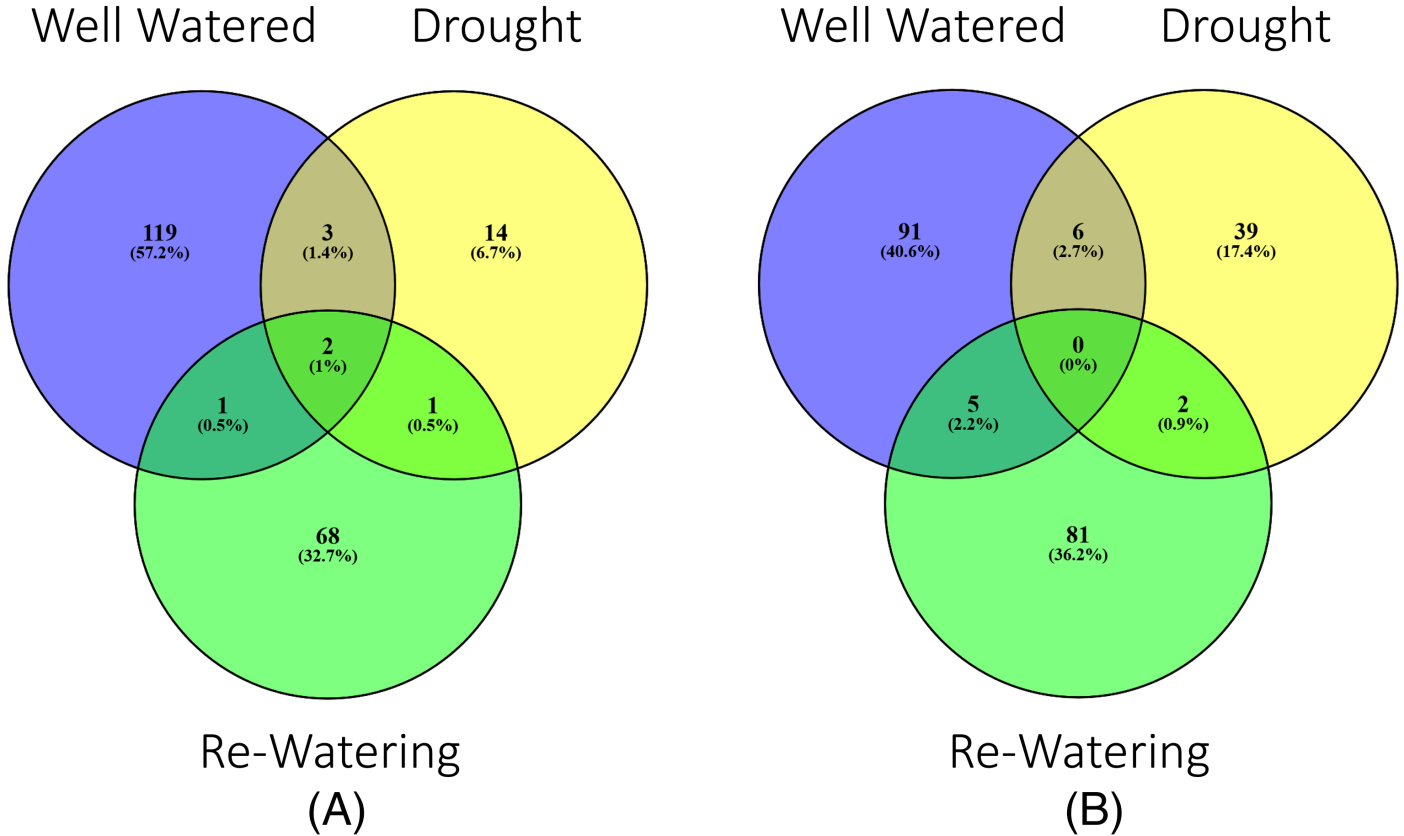
Up‐regulated (A) and down‐regulated (B) genes in *P. aspalathi* “WSF23” inoculated creeping bentgrass roots under well‐watered, drought, and post‐drought recovery conditions, relative to non‐inoculated creeping bentgrass roots (*p* ≤ 0.05).

ACCd bacteria resulted in up‐regulation or down‐regulation of a total of 53 (DEGs) in roots of plants inoculated with the bacteria relative to the non‐inoculated plants under drought stress (Figure 1). Among the up‐regulated differentially‐expressed genes (DEGs) by ACCd bacteria inoculation, 14 genes were found only under drought stress. Inoculation also resulted in three DEGs that were up‐regulated in both drought and well‐watered conditions, two DEGs that were up‐regulated in drought, well‐watered, and re‐watering conditions, and one DEG that was up‐regulated in all three watering treatments (Figure 2A, Table 1, and Table S1). ACCd bacteria caused the down‐regulation of 39 unique DEGs under drought stress. Six DEGs were down‐regulated under both drought and well‐watered conditions, and two DEGs were down‐regulated during both drought and re‐watering (Figure 2B, Table 1, and Table S1).

**TABLE 1:** Uniquely expressed genes in inoculated creeping bentgrass roots after 35 days of drought stress relative to non‐inoculated creeping bentgrass roots (*p* ≤ 0.05).

Up‐regulated			Down regulated		
Accession no.	FC (log2)	Gene name	Accession no.	FC (log2)	Gene name
Os04g0671700	1.18	DJ‐1C	Os03g0202200	−1.69	VDAC5
Os04g0404400	1.15	PCC13‐62	Os01g0363900	−1.53	CRD1
Os05g0426300	1.10	TBL27	Os02g0301000	−1.50	C3H15
Os01g0243200	1.07	Os_F0797	Os03g0597600	−1.45	ASNASE1
Os03g0356414	1.06	DIS1	Os12g0207500	−1.45	ATPASE3
Os07g0638300	1.03	1CYSPRXA	Os01g0533100	−1.39	GSL9
Os03g0254400	1.02	pPLAIIIalpha	Os01g0533000	−1.37	GSL9
Os05g0141400	1.29	Unknown	Os11g0700500	−1.34	MYBAS1
Os03g0133875	1.25	Unknown	Os06g0677000	−1.34	DEP3
Os11g0246900	1.18	Unknown	Os02g0180000	−1.23	PP2C11
Os08g0558100	1.14	Unknown	Os06g0199800	−1.18	GPCR
Os05g0477300	1.09	Unknown	Os09g0563250	−1.14	DHHC23
Os03g0807000	1.06	Unknown	Os02g0639000	−1.12	LMS
Os01g0925200	1.04	Unknown	Os07g0111600	−1.11	PAP9A
			Os09g0346400	−1.10	BIP103
			Os06g0186400	−1.10	SCP32
			Os02g0655500	−1.08	OsSTA68
			Os03g0586900	−1.07	PLS2
			Os02g0181900	−1.01	CLPB‐M
			Os06g0653800	−1.01	DWD71
			Os02g0738600	−1.01	GH9B8
			Os02g0800000	−1.88	Unknown
			Os10g0574800	−1.72	Unknown
			Os08g0562300	−1.45	Unknown
			Os02g0580100	−1.43	Unknown
			Os02g0778400	−1.41	Unknown
			Os06g0106100	−1.34	Unknown
			Os07g0191700	−1.33	Unknown
			Os11g0195100	−1.33	Unknown
			Os03g0645200	−1.28	Unknown
			Os01g0858500	−1.14	Unknown
			Os01g0760400	−1.14	Unknown
			Os01g0812050	−1.12	Unknown
			Os05g0306000	−1.06	Unknown
			Os03g0769000	−1.04	Unknown
			Os03g0170100	−1.03	Unknown
			Os12g0615800	−1.03	Unknown
			Os01g0793500	−1.01	Unknown
			Os03g0388500	−1.00	Unknown

**FIGURE 3:**
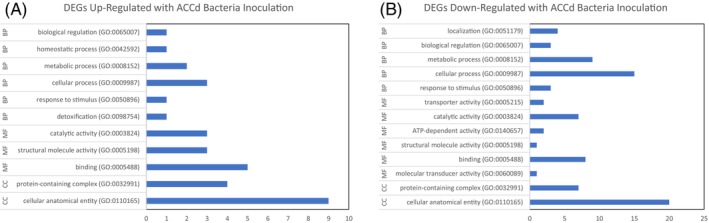
Gene ontology of up‐regulated (A) and down‐regulated (B) genes in *P. aspalathi* “WSF23” inoculated creeping bentgrass roots under drought stress conditions, relative to non‐inoculated creeping bentgrass roots (*p* ≤ 0.05).

The GO Enrichment Analysis demonstrated that up‐regulated DEGs by ACCd bacteria under drought stress included biological processes (biological regulation, homeostatic process, metabolic process, and detoxification), molecular functions (catalytic activity, structural molecule activity, and binding), and cellular components (protein containing complex and cellular anatomical entity) (Figure 3A). Down‐regulated DEGs influenced biological processes (localization, biological regulation, metabolic and cellular processes, and response to stimulus), molecular functions (transporter activity, catalytic activity, ATP‐dependent activity, structural molecular activity, and binding), and cellular components (protein containing complex and cellular anatomical entity) (Figure 3B).

**FIGURE 4:**
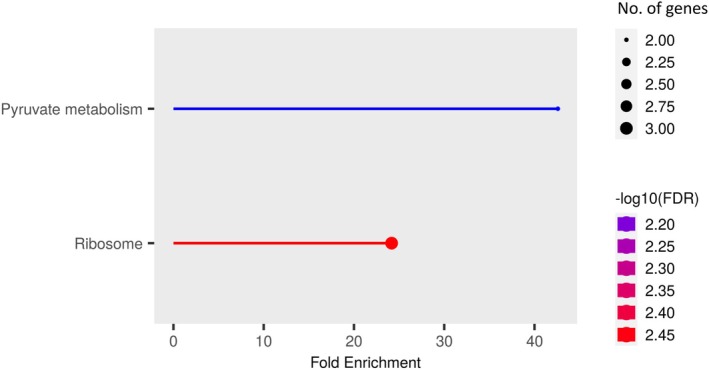
KEGG Pathways associated with up‐regulated DEGs in *P. aspalathi* “WSF23” inoculated creeping bentgrass roots under drought stress conditions, relative to non‐inoculated creeping bentgrass roots (*p* ≤ 0.05).

KEGG Pathway Analysis of the network of gene products, mostly proteins, indicated that the ACCd bacteria up‐regulated genes in drought‐stressed plants that were mainly involved in pyruvate metabolism and ribosome biogenesis (Figure 4).

### 3.2 Differentially Expressed Genes (DEG) Regulated by ACCd Bacteria in Roots of Plants Upon Re‐Watering

Creeping bentgrass roots inoculated with ACCd bacteria demonstrated 160 DEGs after 15 days of re‐watering compared to non‐inoculated plants. There was a total of 72 up‐regulated DEGs and 88 down‐regulated DEGs (Figure 1). Sixty‐eight DEGs were uniquely up‐regulated in inoculated plants upon re‐watering, while one DEG was up‐regulated in both well‐watered and re‐watered treatment groups, in addition to the up‐regulated DEGs in common with drought stress treatments listed above (Figure 2A, Table 2, and Table S2). Eighty‐one DEGs were uniquely down‐regulated in inoculated plants after re‐watering, while five down‐regulated DEGs were common to both re‐watering and well‐watered conditions, in addition to the two down‐regulated DEGs shared between re‐watering and drought stress conditions (Figure 2B, Table 2, and Table S2).

**TABLE 2:** Uniquely expressed genes in inoculated creeping bentgrass roots after post‐drought re‐watering relative to non‐inoculated creeping bentgrass roots (*p* ≤ 0.05).

Up‐regulated			Down‐regulated		
Accession no.	FC (log2)	Gene name	Accession no.	FC (log2)	Gene name
Os04g0667800	2.41	Dhr6	Os01g0868000	−2.07	ERF99
Os01g0746400	2.07	D10	Os09g0468700	−1.89	OsbHLH046
Os03g0292100	1.83	PP2C32	Os09g0437400	−1.82	SAUR38
Os02g0806700	1.67	RING104	Os08g0441100	−1.73	CIPK06
Os02g0652000	1.65	CYCU4	Os01g0281600	−1.66	ENODL4
Os11g0490900	1.55	WRKY72	Os10g0560700	−1.57	ERF36
Os05g0494600	1.49	Os_F0757	Os02g0758800	−1.54	UCL6
Os10g0580400	1.44	DUR3	Os05g0277000	−1.52	EXPA33
Os09g0334500	1.44	WRKY74	Os05g0468800	−1.45	CRP
Os01g0797600	1.41	ERF3	Os04g0581100	−1.39	S3H
Os01g0802100	1.41	GRY340	Os03g0826500	−1.38	ASA1
Os07g0659700	1.38	FBX477	Os07g0105000	−1.37	UCL20
Os04g0655300	1.36	RLCK168	Os06g0682900	−1.36	HSA32
Os02g0681200	1.33	RING327	Os05g0230600	−1.34	RFC3
Os02g0115700	1.33	CATA	Os01g0875300	−1.34	USP7
Os03g0820300	1.32	ZFP182	Os02g0699700	−1.33	TOP2
Os04g0556000	1.31	HMA5	Os08g0253800	−1.32	CSLC3
Os04g0578400	1.27	BCH2, HYD1	Os08g0327400	−1.31	ENR1
Os10g0399200	1.27	CGS1	Os05g0572000	−1.30	RPH1
Os01g0147200	1.25	ASD1	Os03g0682100	−1.29	MFS1
Os07g0190000	1.22	DXS3	Os01g0644200	−1.28	SALP1
Os03g0838800	1.22	HINGE3	Os04g0473400	−1.28	RPL6
Os02g0681700	1.22	RLCK83	Os03g0297100	−1.24	RPS7A
Os02g0282900	1.20	ABCE1	Os01g0273100	−1.22	UBC27
Os03g0242900	1.19	SUB28	Os10g0565150	−1.22	BMY6
Os05g0556100	1.15	DRP1A	Os11g0591100	−1.21	GME2
Os03g0744650	1.12	WD40‐90	Os04g0531100	−1.21	RPP16
Os07g0225300	1.11	NAC3	Os03g0822700	−1.20	FES1A
Os05g0530400	1.09	SPL7	Os05g0399300	−1.20	CHT2
Os01g0667600	1.09	RAB11C1	Os10g0417600	−1.17	GME1
Os08g0118000	1.08	AHL1	Os12g0582800	−1.16	OSCA2.4
Os03g0349200	1.08	OsCDK2	Os06g0561000	−1.15	MIOX
Os04g0645100	1.07	FLO2	Os01g0675100	−1.14	PRXIIC
Os03g0741100	1.07	BHLH148	Os01g0801500	−1.13	GNS7
Os07g0150700	1.06	CIPK23	Os02g0797400	−1.13	MCM5
Os02g0114000	1.05	BRM	Os01g0905700	−1.13	RING204
Os05g0354400	1.04	XOAT5	Os01g0152900	−1.10	H2B.7
Os03g0197800	1.04	IDD1	Os08g0509100	−1.09	LOX8
Os04g0444200	1.04	CAM	Os01g0622300	−1.08	HEME
Os09g0566550	1.03	CTR1	Os05g0437100	−1.07	ERF105
Os06g0192800	1.03	ATL69	Os05g0274200	−1.07	MSH2
Os01g0202500	1.02	BBX1	Os01g0153300	−1.06	H2B.5
Os05g0426200	1.02	NAC36	Os03g0606200	−1.06	RMtATP6
Os01g0194000	2.27	Unknown	Os12g0133050	−1.06	OsP0C
Os04g0667850	1.99	Unknown	Os01g0668100	−1.05	FLA7
Os07g0164800	1.87	Unknown	Os10g0390500	−1.04	ALAAT
Os08g0427900	1.70	Unknown	Os04g0441800	−1.02	PTR
Os04g0421800	1.63	Unknown	Os08g0508800	−1.02	LOX2
Os03g0263900	1.59	Unknown	Os05g0418100	−1.01	MLO_
Os01g0570500	1.56	Unknown	Os03g0721900	−2.07	Unknown
Os08g0540300	1.52	Unknown	Os01g0205500	−1.78	Unknown
Os02g0177800	1.41	Unknown	Os08g0112566	−1.77	Unknown
Os08g0109000	1.40	Unknown	Os03g0162200	−1.65	Unknown
Os07g0569166	1.39	Unknown	Os03g0685500	−1.63	Unknown
Os03g0349000	1.34	Unknown	Os01g0839300	−1.61	Unknown
Os01g0148050	1.27	Unknown	Os04g0635500	−1.53	Unknown
Os04g0408600	1.25	Unknown	Os01g0969100	−1.47	Unknown
Os02g0802400	1.19	Unknown	Os08g0151400	−1.41	Unknown
Os02g0626532	1.19	Unknown	Os08g0490800	−1.41	Unknown
Os05g0183900	1.17	Unknown	Os01g0502900	−1.38	Unknown
Os02g0177900	1.12	Unknown	Os05g0555800	−1.34	Unknown
Os04g0565200	1.11	Unknown	Os02g0189800	−1.34	Unknown
Os01g0764900	1.09	Unknown	Os05g0394200	−1.33	Unknown
Os01g0708600	1.08	Unknown	Os08g0130550	−1.22	Unknown
Os12g0163700	1.07	Unknown	Os02g0236000	−1.20	Unknown
Os01g0558850	1.06	Unknown	Os07g0656800	−1.19	Unknown
Os03g0219300	1.03	Unknown	Os12g0481400	−1.16	Unknown
Os06g0591600	1.03	Unknown	Os10g0418000	−1.15	Unknown
			Os01g0152700	−1.05	Unknown
			Os05g0466600	−1.03	Unknown
			Os03g0628900	−1.00	Unknown

**FIGURE 5:**
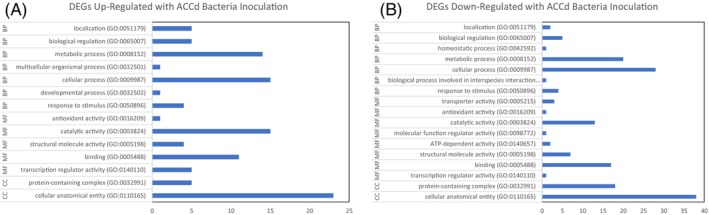
Gene ontology of up‐regulated (A) and down‐regulated (B) genes in *P. aspalathi* “WSF23” inoculated creeping bentgrass roots upon re‐watering, relative to non‐inoculated creeping bentgrass roots (*p* ≤ 0.05).

The GO Enrichment Analysis indicated DEGs up‐regulated by ACCd bacteria during re‐watering influenced the regulation of biological processes including localization, biological regulation, metabolic process, multicellular organismal process, cellular process, developmental process, and response to stimulus, as well as molecular functions including antioxidant activity, catalytic activity, structural molecule activity, binding, and transcription regulator activity, and the cellular components for protein‐containing complex and cellular anatomical entity (Figure 5A). DEGs down‐regulated by ACCd bacteria inoculation during re‐watering influenced the biological processes of localization, biological regulation, homeostatic process, and metabolic and cellular processes. Biological processes involved in interspecies interactions between organisms, and response to stimulus, as well as molecular functions of transporter activity, antioxidant activity, catalytic activity, molecular function regulator activity, ATP‐dependent activity, structural molecular activity, binding, and transcription regulator activity, and cellular components of protein‐containing complex, and cellular anatomical entity were also affected (Figure 5B).

**FIGURE 6:**
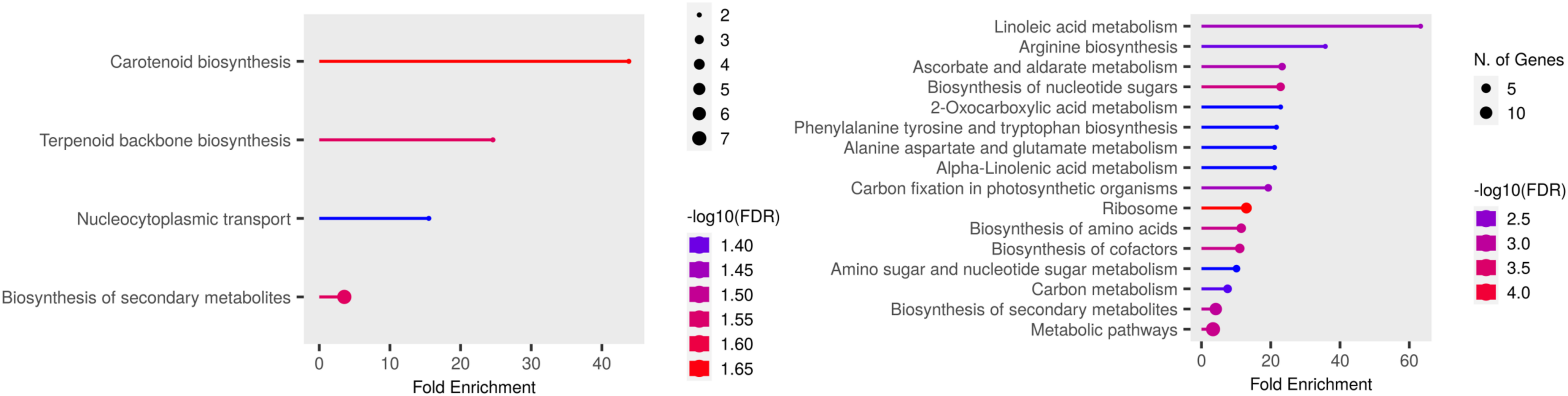
KEGG Pathways associated with up‐regulated (A) and down‐regulated (B) DEGs in *P. aspalathi* “WSF23” inoculated creeping bentgrass roots upon re‐watering, relative to non‐inoculated creeping bentgrass roots (*p* ≤ 0.05).

The KEGG Pathway Analysis revealed that DEGs up‐regulated by ACCd bacteria during re‐watering mainly influenced carotenoid biosynthesis, terpenoid backbone biosynthesis, nucleocytoplasmic transport, and biosynthesis of secondary metabolites. DEGs down‐regulated by inoculation affected linoleic acid metabolism, arginine biosynthesis, ascorbate and aldarate metabolism, and biosynthesis of nucleotide sugars (Figure 6).

## 4 Discussion

**FIGURE 7:**
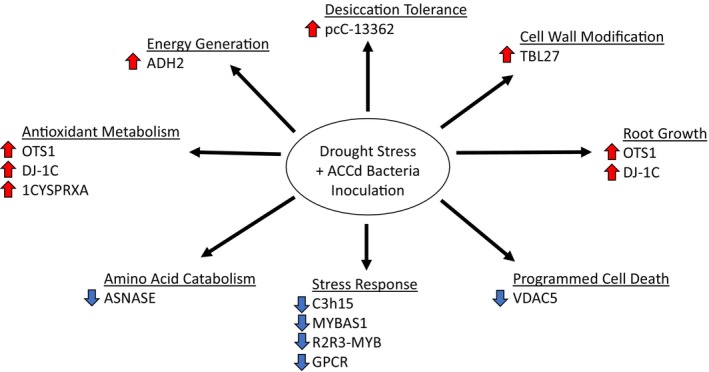
Summary of genes and associated metabolic pathways regulated by *P. aspalathi* “WSF23” contributing to improved drought tolerance. The upward arrows indicate up‐regulated genes by the inoculation. The downward arrows indicate down‐regulated genes by the inoculation.

**FIGURE 8:**
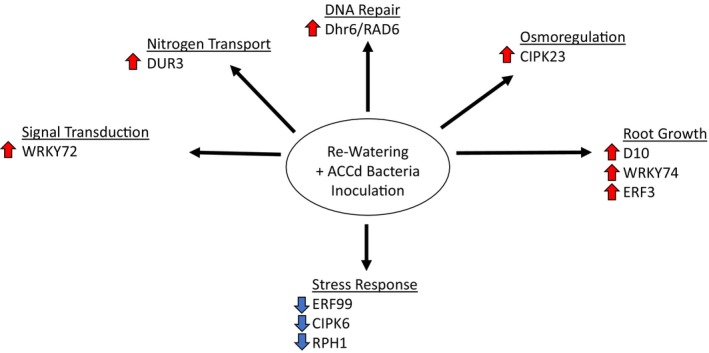
Summary of genes and associated metabolic pathways regulated by *P. aspalathi* “WSF23” contributing to improved post‐stress recovery. The upward arrows indicate up‐regulated genes by the inoculation. The downward arrows indicate down‐regulated genes by the inoculation.

Improved drought tolerance by ACCd bacteria has been associated with improvement in physiological and metabolic activities (Saikia et al. 2018; Naing et al. 2021; Errickson et al. 2023; Errickson and Huang 2023; Shahid et al. 2024). This study found that improved plant tolerance to drought stress and post‐stress recovery by the ACCd bacteria, *P. aspalathi* “WSF23,” was associated with differential transcriptional regulation in the roots of creeping bentgrass under drought stress and upon re‐watering. Specific genes regulated by *P. aspalathi* “WSF23” under drought stress and re‐watering conditions are summarized in the pathway maps (Figures 7 and 8). Selected genes with known functions regulated by *P. aspalathi* “WSF23” under drought stress or during re‐watering that may be related to stress tolerance and recovery are discussed below.

### 4.1 ACCd Bacteria Regulated Genes and Associated Cellular and Metabolic Pathways for Improving Drought Tolerance

Among all the up‐regulated genes, *OVERLY TOLERANT TO SALT 1* (*OTS1*) demonstrated the highest fold‐change (by 1.6 FC) in plants inoculated with *P. aspalathi* “WSF23” exposed to drought stress. *OTS1* codes for SUMO (Small Ubiquitin‐Like Modifier) protease, which controls filament elongation and cell expansion or elongation and plays key roles in root development and seed germination, as well as plant tolerance to abiotic stress, including drought and salt stress (Srivastava et al. 2016). Overexpression of *OTS1* in sugarcane ( *Saccharum* spp. hybrids), which is another perennial grass species, similarly resulted in improved drought tolerance, with plants demonstrating higher stomatal conductance, chlorophyll content, and photosynthesis rate, as well as lower protease activity in *OTS1* overexpression plants subjected to drought stress (Masoabi et al. 2023). Overexpression of *OTS1* in winter wheat ( *Triticum aestivum* ) enhanced root growth, chlorophyll content, and antioxidant activity under drought stress conditions (Le Roux et al. 2019). Overexpressing *OTS1* in rice increased root length and fresh weight in plants exposed to salt stress (Srivastava et al. 2016). The alcohol dehydrogenase gene, *ADH2*, was the second‐highest up‐regulated gene in inoculated roots during drought stress (by 1.2 FC). *ADH2* codes for alcohol dehydrogenase, which oxidizes alcohols to generate energy for cellular activities and detoxifies cellular damage from the accumulation of alcohol under anaerobic conditions. *ADH2* may be induced by soil dry down during drought stress and has conferred abiotic stress resistance in various plant species (Shi et al. 2017; Wang et al. 2024; Zhang et al. 2024). The increased transcript levels of *OTS1* and *ADH2* following ACCd bacteria inoculation suggest that stress defense systems are activated to protect against cellular damage from dehydration and alcohol to maintain root growth and whole‐plant tolerance to drought stress (Errickson et al. 2023).

*P. aspalathi* “WSF23” inoculation led to significant upregulation of *DJ‐1C* under drought stress. This gene belongs to the DJ‐1 superfamily of nuclear gene‐encoding proteins (Lin et al. 2011). *DJ‐1C* has diverse biological functions, including plastid development and protection against oxidative stress, as well as stress responses (Lin et al. 2011; Wang, Shao, et al. 2023). DJ‐1 proteins have also been shown to contribute to reducing oxidative stress in Arabidopsis by interacting with superoxide dismutase 1 (SOD1) and glutathione peroxidase 2 (GPX2) (Xu et al. 2010; Lin et al. 2011). The improvement of drought and salinity stress tolerance in rice associated with the positive expression of *DJ‐1C* has been attributed to enhancing root growth, photosynthesis, antioxidant activity, and redox homeostasis (Rathore et al. 2024). Up‐regulation of another gene, *1CYSPRXA*, by *P. aspalathi* “WSF23” inoculation also likely contributed to a reduction in ROS. This gene codes for a peroxiredoxin, which is an antioxidant that reduces alkyl hydroperoxides to alcohols and hydrogen peroxide to water, protecting lipids, enzymes, and DNA from oxidative damage (Lee et al. 2000; Haslekas et al. 2003; Kim et al. 2011). Our results, combined with others, suggest that *DJ‐1C* and *1CYSPRXA* could play important roles in ACCd bacteria‐regulated drought tolerance relating to oxidative stress defenses induced by drought stress, among other effects.

Desiccation‐related protein (DRP) gene, *pcC‐13362*, was also up‐regulated in inoculated roots subjected to drought stress. The LEA‐like DRP pcC‐13362 has been found to accumulate abundantly in tissues of highly desiccation‐tolerant species such as the resurrection plant *raterostigma plantagineum* and its close relative *Lindera brevidens*, while only accumulating to lower levels in the desiccation‐sensitive species *Lindera subracemosa* (Giarola et al. 2018). The DRP pcC‐13362 has also been identified as a key protein in drought‐tolerant cultivars of the bean ( *Phaseolus vulgaris* L.) (Pérez et al. 2024) and is up‐regulated in response to pathogen infection (Piatkowski et al. 1990). *TBL27* was also up‐regulated (by 1.1 FC) by inoculation during drought stress. This gene is involved in the acetylation of xyloglucan in cell walls, which is important for the structure and elasticity of cell walls (Gille et al. 2011; Zhu et al. 2014; Daher et al. 2024), which contributes to improved desiccation tolerance (Moore et al. 2008). The up‐regulation of *pcC‐13362* and *TBL27* by *P. aspalathi* “WSF23” inoculation may lead to increased drought tolerance in creeping bentgrass, involving the maintenance of cell wall elasticity, which plays a critical role in protecting cells from dehydration during drought stress.

The KEGG Pathway Analysis indicated that pyruvate metabolism was the most positively enriched metabolic pathway related to up‐regulated DEGs in inoculated plants after 35 days of drought stress. Pyruvate's role in cellular energy metabolism (Fink 2007; McCommis and Finck 2015) has been well known, and recent studies have further demonstrated the role of pyruvate in promoting abiotic stress tolerance through acting as an antioxidant, encouraging expression of antioxidant enzymes, and up‐regulating stress‐responsive genes and transcription factors during abiotic stress (Alam et al. 2025). Furthermore, KEGG analysis also demonstrated that DEGs related to ribosome biosynthesis were highly up‐regulated in drought‐stressed plants due to ACCd bacteria inoculation. Ribosome biogenesis is responsible for the generation of ribosomes for protein synthesis in cells, which play positive roles in plant tolerance to drought and salt stress (Shiraku et al. 2021). These results suggest that *P. aspalathi* “WSF23” inoculation could enhance root growth and improve drought tolerance of creeping bentgrass (Errickson et al. 2023) through up‐regulating genes for sustained energy and protein metabolism, along with enhanced stress defense via the enrichment of the pyruvate metabolism pathway and ribosome biogenesis.

ACCd bacteria inoculation also resulted in the down‐regulation of some DEGs under drought stress, including some transcriptional factors or genes involved in stress responses (*C3H15* by −1.5 FC, *MYBAS1* by −1.3 FC, *R2R3‐MYB* by −1.2 FC, and *GPCR* by −1.2 FC) or stress‐induced programmed cell death (*VDAC5* by −1.69 FC). *C3H15* codes for a CCCH zinc‐finger protease. Previous RT‐qPCR studies have found that these CCCH zinc‐finger protease genes are induced by drought stress and ABA levels in plants under stress (Han et al. 2021). *MYBAS1* and *R2R3‐MYB* are involved in ABA‐mediated leaf senescence (Guo et al. 2017) and have been associated with reduced biomass (Fávero Peixoto‐Junior et al. 2018). MYBs exhibited lower expression in a drought‐tolerant rice genotype relative to that in a drought‐susceptible genotype (Nawae et al. 2020). *GPCR*, encoding a G‐coupled protein receptor, was also down‐regulated in inoculated roots under drought stress conditions relative to non‐inoculated controls. GPCRS are membrane proteins that are important for signal transduction, with G proteins being induced in response to ABA and other abiotic stresses (Nitta et al. 2015). This corresponds to our previous work, in which ABA levels were lower in creeping bentgrass inoculated with *P. aspalathi* compared to non‐inoculated controls (Errickson et al. 2023), suggesting that stress‐responsive genes induced by ABA may be down‐regulated as a result of inoculation. *VDAC5* was also down‐regulated and is a member of the VDAC family, which comprises voltage‐dependent anion channels located on the membrane of the mitochondria. VDACs have been implicated in programmed cell death induced by abiotic stress, including drought (Godbole et al. 2003, 2013; Desai et al. 2006; Homblé et al. 2012). These results suggest that plants colonized by *P. aspalathi* “WSF23” may experience a lesser extent of drought stress damage as demonstrated by the down‐regulation of stress‐responsive transcriptional factors or genes associated with stress‐induced cell death, while genes associated with stress protection were up‐regulated.

*P. aspalathi* “WSF23” inoculation also resulted in the down‐regulation of genes associated with amino acid catabolism, such as *ASNASE1* (by −1.45 FC). *ASNASE1*, coding for asparaginase, was down‐regulated with *P. aspalathi* inoculation under drought stress. Reduced asparaginase activity also correlates with the increased asparagine levels observed in drought‐stressed creeping bentgrass inoculated with *P. aspalathi* “WSF23” (Errickson and Huang 2023). Our results indicated that the suppression of asparagine catabolism by *P. aspalathi* inoculation could contribute to the maintenance of this important amino acid to support plant growth under drought stress for creeping bentgrass.

### 4.2 ACCd Bacteria Regulated Genes and Associated Cellular and Metabolic Pathways for Improving Post‐Drought Recovery Upon Re‐Watering

Our previous studies found that *P. aspalathi* “WSF23” inoculation resulted in more rapid growth recovery of creeping bentgrass from drought stress upon re‐watering (Errickson et al. 2023). In this study, several DEGs associated with plant growth were more highly expressed in plants inoculated with *P. aspalathi* “WSF23” upon re‐watering, following the drought stress period. *Dhr6/RAD6*, which encodes a ubiquitin‐conjugating enzyme associated with DNA repair and proliferation of new growth (Yamamoto et al. 2004), was up‐regulated by 2.4 FC, and *D10*, encoding a carotenoid cleavage dioxygenase and supporting strigolactone (SL) biosynthesis, was up‐regulated by 2.1 FC. D10 is involved in the regulation of lateral growth, including tillering and lateral root growth, with localization in the parenchyma of root cells in the stele and xylem parenchyma in the stem. D10 has been shown to stimulate SL biosynthesis, influencing root architecture (Kumar et al. 2015; Sun et al. 2015) and cytokinin biosynthesis that affects tiller production and leaf senescence (Zhang et al. 2010; Ding et al. 2024). These findings are consistent with previous work that has demonstrated increased cytokinin levels and enhanced tiller production in creeping bentgrass inoculated with *P. aspalathi* “WSF23,” which may ultimately contribute to a more rapid recovery upon re‐watering (Errickson et al. 2023). Carotenoid biosynthesis was also identified by KEGG analysis involving up‐regulated DEGs following 15 days of re‐watering. Among the many roles that carotenoids play in plant growth and development, carotenoids and their derivatives have been shown to affect root architecture and adaptation to the rhizosphere environment, including initiation of lateral roots (Ke et al. 2022), further supporting previous evidence of improved root growth with *P. aspalathi* inoculation (Errickson et al. 2023).

Several *WRKY* genes were also upregulated during re‐watering, including *WRKY72* (by 1.55 FC) and *WRKY74* (by 1.4 FC). *WRKY72* can be induced by JA, ABA, and bacterial infection and plays a role in abiotic stress signaling (Ashwini et al. 2016). *WRKY74* plays a positive role in plant tolerance to low inorganic phosphate (Pi) stress and root growth under low Pi conditions, and is inducible by bacterial inoculation (Dai et al. 2016). Improved post‐stress recovery in creeping bentgrass inoculated with *P. aspalathi* “WSF23” could also have resulted from up‐regulation of *DUR3* (by 1.4 FC), which is a urea transporter that plays a role in root uptake of urea (Wang et al. 2012; Beier et al. 2019). *ERF3* (+1.3 FC) was also up‐regulated in inoculated roots during re‐watering. ERF3 is essential for crown root development and functions in auxin and cytokinin‐responsive gene expression and signaling (Zhao et al. 2015). *CIPK23* (+1.1 FC) encodes a CBL‐interacting protein kinase that activates K^+^ transporters to facilitate ion homeostasis and increased K^+^ uptake, which may have also contributed to increased root growth upon re‐watering and enhanced osmoregulation, with K^+^ serving as a compatible solute (Ragel et al. 2015). Accumulation of the compatible solute, proline, has also been associated with *P. aspalathi* inoculation (Errickson and Huang 2023).

In response to re‐watering, several stress‐responsive genes were down‐regulated by ACCd bacteria inoculation of roots. *ERF99*, an ethylene response factor that is induced by abiotic stress (Balfagón et al. 2020), was down‐regulated by 2.1 FC in inoculated plants during re‐watering. This may be a result of *P. aspalathi* “WSF23” limiting ethylene production to reduce the expression of stress‐responsive ERFs, as reductions in ethylene have been previously reported (Errickson et al. 2023). Another stress‐inducible DEG, *CIPK6*, was also down‐regulated (1.7 FC) in inoculated plants. *CIPK6* is expressed during drought and salinity stress, and with increased ABA concentrations (Chen et al. 2013), which is consistent with our previous findings of reduced ABA in inoculated plants (Errickson et al. 2023). *RPH1* is a transcriptional repressor that regulates stress‐responsive genes and was down‐regulated (1.3 FC) in inoculated plants. Oxidative stress and DNA damage induce phosphorylation of Rph1, which leads to activation of downstream stress‐activated genes (Liang et al. 2013). The downregulation of *RPH1* is further evidence of reduced oxidative stress or DNA damage in plants inoculated with *P. aspalathi* “WSF23.” KEGG analysis identified linoleic acid metabolism as the pathway most affected by ACCd bacteria inoculation, involving down‐regulated DEGs in this pathway during re‐watering. Linoleic acid plays a diverse range of roles in regulating plant growth and development. It can be converted into signaling molecules like jasmonic acid, which is involved in multiple signaling pathways for plant defense responses against stress (Savchenko et al. 2014). The reduced expression of DEGs associated with linoleic acid metabolism may be indicative of inoculated plants rapidly changing from stress defense mode to regrowth upon re‐watering.

## 5 Conclusions

ACCd‐bacteria, *P. aspalathi* “WSF23,” improved drought tolerance and post‐stress recovery in creeping bentgrass could be associated with transcriptional regulation in the root system. The up‐regulation of genes affecting antioxidant metabolism (*OTS1*, *DJ‐1C*, *1CYSPRXA*), energy generation (*ADH2*), desiccation tolerance (*pcC‐13362*), cell wall modification (*TBL27*), and root growth (*OTS1*, *DJ‐1C*), as well as pathways involved in pyruvate metabolism and ribosome biogenesis, could contribute to improved drought tolerance. ACCd‐bacteria promoted post‐stress recovery could be attributed to the up‐regulation of genes regulating DNA repair (*RAD6*), root development (*D10*, *WRKY74*, *ERF3*), nitrogen transport (*DUR3*), signal transduction (*WRKY72*), and osmoregulation (*CIPK23*), as well as pathways involved in carotenoid biosynthesis. These findings suggest novel mechanisms by which the ACCd bacteria *P. aspalathi* can promote drought stress tolerance and post‐drought recovery in cool‐season grass species, which may contribute to sustainable methods of reducing water use in turfgrass management. Future work may investigate gene–gene and gene‐protein interactions in cool‐season turfgrass inoculated with ACCd bacteria to further develop a mechanistic understanding of bacteria‐mediated improvement of drought tolerance.

## Author Contributions

W.E. conducted the experiment, data analysis, and wrote the manuscript. B.H. developed the research ideas, experimental design, acquired funding, and revised the manuscript.

## Funding

This work was supported by Center for Turfgrass Science New Jersey Agricultural Experiment Station Rutgers, The State University of New Jersey 59 Dudley Rd. New Brunswick, NJ 08901‐8520.

## Conflicts of Interest

The authors declare no conflicts of interest.

## Supporting information


**Table S1:** Differentially expressed genes resultant from inoculation found in drought stressed plants and across other irrigation treatments relative to non‐inoculated roots (*p* ≤ 0.05).
**Table S2:** Differentially expressed genes resultant from inoculation found after re‐watering plants following 35 days of drought stress that were also differentially expressed across other irrigation treatments relative to non‐inoculated roots (*p* ≤ 0.05).

## Data Availability

The data that support the findings of this study are available on request from the corresponding author. The data are not publicly available due to privacy or ethical restrictions.
