# Supplementary material for: Transcriptional Regulation in Roots by Bacteria With 1‐Aminocyclopropane‐1‐Carboxylate Deaminase Enzymes for Drought Tolerance and Post‐Stress Recovery
Source: Physiol Plant. 2026 Jan 2;178(1):e70733. doi: 10.1111/ppl.70733 (PMC12757826; doi:10.1111/ppl.70733)
Supplement: Supplementary file 1 — Table S1: Differentially expressed genes resultant from inoculation found in drought stressed plants and across other irrigation treatments relative to non‐inoculated roots (p ≤ 0.05). Table S2: Differentially expressed genes resultant from inoculation found after re‐watering plants following 35 days of drought stress that were also differentially expressed across other irrigation treatments relative to non‐inoculated roots (p ≤ 0.05). [file PPL-178-e70733-s001.docx]

Supplementary Table 1: Differentially expressed genes resultant from inoculation found in drought stressed plants and across other irrigation treatments relative to non-inoculated roots (P≤ 0.05).

| **Up-Regulated** |  |  |
| --- | --- | --- |
| **Accession no.** | **FC (log2)** | **Gene Name** |
| **Drought, Well Watered, and Re-Watering** |  |  |
| Os06g0487900 | 1.58 | OTS1 |
| Os07g0674700 | 1.14 | unknown |
|  |  |  |
| **Drought and Well-Watered** |  |  |
| Os02g0194200 | 1.23 | C3H14 |
| Os11g0210500 | 1.16 | ADH2 |
| Os08g0556600 | 1.15 | unknown |
|  |  |  |
| **Drought and Re-Watering** |  |  |
| Os05g0459900 | 1.11 | SPR9 |
|  |  |  |

| **Down-Regulated** |  |  |
| --- | --- | --- |
| **Accession no.** | **FC (log2)** | **Gene Name** |
| **Drought and Well-Watered** |  |  |
| Os01g0725800 | -1.22 | WD40-24 |
| Os12g0567300 | -1.15 | R2R3-MYB |
| Os02g0749300 | -1.04 | SK1 |
| Os03g0356484 | -2.13 | unknown |
| Os05g0402851 | -1.36 | unknown |
| Os09g0413600 | -1.21 | unknown |
|  |  |  |
| **Drought and Re-Watering** |  |  |
| Os11g0433900 | -1.08 | RPL38 |
| Os06g0496000 | -1.07 | unknown |

Supplementary Table 2: Differentially expressed genes resultant from inoculation found after re-watering plants following 35 d of drought stress that were also differentially expressed across other irrigation treatments relative to non-inoculated roots (P≤ 0.05).

| **Up-Regulated** |  |  |
| --- | --- | --- |
| **Accession no.** | **FC (log2)** | **Gene Name** |
| **Re-Watering, Drought, and Well-Watered** |  |  |
| Os06g0487900 | 2.65 | OTS1 |
| Os07g0674700 | 1.48 | unknown |
|  |  |  |
| **Re-Watering and Well-Watered** |  |  |
| Os08g0495500 | 1.98 | C3-BTB2 |
|  |  |  |
| **Re-Watering and Drought** |  |  |
| Os05g0459900 | 1.01 | SPR9 |

| **Down-Regulated** |  |  |
| --- | --- | --- |
| **Accession no.** | **FC (log2)** | **Gene Name** |
| **Re-Watering and Well-Watered** |  |  |
| Os03g0424500 | -1.53 | RPS4 |
| Os03g0226200 | -1.03 | Hb2 |
| Os01g0926300 | -1.53 | unknown |
| Os07g0546700 | -1.39 | unknown |
| Os06g0714100 | -1.10 | unknown |
|  |  |  |
| **Re-Watering and Drought** |  |  |
| Os11g0433900 | -1.40 | RPL38 |
| Os06g0496000 | -1.07 | unknown |
